# Prophylactic effect of nano vitamin D3 against fatty degeneration caused by calcium-deficient diet in parotid salivary gland: a histological and ultrastructural study

**DOI:** 10.1186/s12903-025-06618-7

**Published:** 2025-07-30

**Authors:** Maha Mohamed Montaser, Dina M. Mahdy, Sara A. Hamza

**Affiliations:** 1 College of Dentistry, Arab Academy for Science, Technology and Maritime Transport, Alamein Campus, New Al-Alamein City, Egypt; 2 College of Pharmacy, Arab Academy of Science and Technology and Maritime Transport, Alamein Campus, New Al-Alamein City, Egypt; 3 Faculty of Dentistry, Alexandria University, Alexandria, Egypt

**Keywords:** Calcium, Parotid salivary gland, Calcium-deficient diet, Conventional vitamin D3, Vitamin D3-loaded lipid nano capsules (LNCs), Secretory granules

## Abstract

**Background:**

Poor dietary habits, such as a calcium-deficient diet, negatively impact oral health by impairing salivary gland function due to hypocalcemia. If left untreated, calcium deficiency can lead to fatal consequences. Several nano-formulations, including nano vitamin D3 supplements, have recently been introduced. However, there is limited research on the impact of vitamin D3-loaded lipid nano capsules (LNCs) on dental health maintenance.

**Aim:**

This study evaluated the protective potential of vitamin D3-loaded lipid nano capsules (LNCs) versus conventional vitamin D3 in countering fatty degeneration in parotid salivary glands caused by a calcium-deficient diet.

**Methods:**

Thirty-six male mature albino rats were randomly split into three groups: a calcium-deficient group, a calcium-deficient group treated with conventional vitamin D3, and another receiving vitamin D3-loaded lipid nano capsules (LNCs). The body weight was calculated weekly throughout the experimental duration. At the end of the experiment, blood samples were obtained for serological analysis of serum calcium and insulin concentration as well as blood glucose levels. Parotid glands were harvested for histological and ultrastructural examination. Digital morphometry was used to assess the number of secretory granules. All the data was collected and statistically analyzed using one-way ANOVA and Tukey’s honestly significant difference test.

**Results:**

The conventional vitamin D3 group showed an incomplete protective effect, while vitamin D3-loaded lipid nano capsules (LNCs) completely preserved cellular structures, preventing the salivary tissue’s fatty deterioration. Vitamin D3-loaded lipid nano capsules (LNCs) outperformed conventional vitamin D3 when preserving the quantity of secretory granules.

**Conclusion:**

Supplementing calcium-deficient diets with vitamin D3-loaded lipid nano capsules (LNCs) could protect against salivary gland damage caused by calcium deficiency in rats.

## Introduction

Most people are aware of calcium’s vital function in preserving bone health and averting osteoporosis. Besides strengthening bones and teeth, calcium is essential for several physiological functions. It participates in cell-signaling pathways as a messenger, facilitates normal cell operations, aids in nerve signal transmission, supports hormone secretion, calcium is vital for blood clotting, and is necessary for muscle contraction and relaxation. These roles underscore the importance of sufficient calcium levels for overall health and bodily functions [1, 2]. The smooth endoplasmic reticulum (SER) stores calcium at the cellular level, playing a key role in various cellular processes. It is also utilized by the nucleus and in the mitochondria. Additionally, calcium is crucial for adherent junctions and tight junctions [3].

A calcium deficiency (hypocalcemia) disrupts essential calcium-dependent processes in these organelles. In the nucleus, it impairs gene transcription, cell cycle progression, and apoptosis regulation. In the mitochondria, reduced calcium levels can compromise ATP production, disrupt membrane potential, and increase oxidative stress through elevated reactive oxygen species (ROS). In the SER, hypocalcemia interferes with calcium signaling and protein folding, activating endoplasmic reticulum stress and the unfolded protein response (UPR), which can ultimately result in cellular dysfunction or apoptosis [4].

One underexplored area is how hypocalcemia affects the salivary glands, particularly their structure and function. Calcium is involved in salivary secretion through its role in neurotransmitter signaling and intracellular pathways, including regulation of PKA, PKC, and cAMP levels [5]. Calcium also controls the opening of ion and water channels, such as potassium, chloride, and aquaporins, essential for fluid movement in acinar cells [6, 7]. It facilitates secretory granule formation and exocytosis by promoting protein condensation and membrane fusion. Therefore, a calcium deficiency may contribute to xerostomia by impairing salivary secretion, increasing immature secretory granules, and disrupting glandular homeostasis [8]. Moreover, hypocalcemia can worsen insulin resistance (IR), which further impairs salivary gland function by reducing saliva production, promoting inflammation, and disrupting metabolic pathways [9].

Given the detrimental effects of hypocalcemia on both cellular organelles and salivary gland function, identifying effective interventions to restore calcium balance and preserve glandular integrity is essential. One such intervention is vitamin D supplementation, which plays a pivotal role in calcium homeostasis and insulin regulation.

Vitamin D, a fat-soluble vitamin, plays a central role in correcting hypocalcemia by promoting calcium absorption and regulating insulin sensitivity. It exists in two forms—vitamin D2 (ergocalciferol) from plant sources and vitamin D3 (cholecalciferol) from animal sources or synthesized in the skin through sunlight exposure. Both are converted into the active form, calcitriol, in the liver and kidneys [10–12].

When it comes to increasing serum vitamin D levels, vitamin D3 is thought to be more effective than D2 [13]. However, vitamin D insufficiency is frequent and linked to chronic illnesses because of inadequate dietary intake and limited sun exposure. Conditions such as cardiovascular disease, obesity, fatty liver, and autoimmune disorders are all related to vitamin D deficiency. Supplementation with vitamin D can improve insulin sensitivity and help mitigate hypocalcemia-related complications [14].

Recent advancements have focused on enhancing the bioavailability and stability of vitamin D through nanotechnology-based delivery systems. Compared to ordinary emulsion vitamin D, nano vitamin D dissolves more easily in the intestines [15]. It is also more efficiently absorbed and put into circulation, leading to higher tissue bioavailability [16]. Compared to conventional vitamin D, nano vitamin D can raise blood vitamin levels by almost three times [17]. The effective form of vitamin D acts like a hormone. In addition to its main role in preserving calcium and phosphorus amounts in the blood, it takes part in many physiological processes, such as regulating the immune response and influencing cell growth and differentiation [11].

Lipid nano capsules (LNCs) are core-shell structures with a triglyceride core and a surfactant membrane, offering stable and controllable drug delivery [18]. Made in a single step using phase inversion temperature, they can carry both lipophilic and hydrophilic substances and support extended-release formulations [19, 20] Encapsulation protects vitamin D3 from degradation by light and oxygen, improves shelf life, and enhances controlled release [21].

A recent study by Shakeri et al. (2024) showed that beeswax solid lipid nanoparticles effectively encapsulate vitamin D3 and omega-3, offering high encapsulation efficiency under stress conditions [20, 21]. Despite these promising findings, there is limited research comparing the effectiveness of vitamin D3 D3-loaded lipid nano capsules to conventional vitamin D3 in protecting salivary glands from calcium-deficiency-induced damage [22, 23].

This study aims to evaluate and compare the histological effects of vitamin D3-loaded lipid nano capsules (LNCs) and commercially available vitamin D3 in preventing salivary gland degeneration caused by a calcium-deficient diet in male albino rats.

The null Hypothesis states that conventional vitamin D3 and vitamin D3-loaded lipid nano capsules (LNCs) do not significantly differ in preventing the effects of a calcium-deficient diet on the parotid salivary gland.

This study addresses a critical gap in the literature by comparing the effects of conventional and nano-formulated vitamin D3 on salivary gland structure and systemic biochemical parameters. While previous research has focused mainly on the systemic roles of vitamin D3, little is known about its direct impact on salivary glands, particularly in calcium-deficient conditions. Filling this gap through comprehensive evaluation—including histological, ultrastructural, morphometric, and biochemical analyses—this study aims to determine the differential effects of these two forms of vitamin D3 and answer the central question: how do conventional and nano-formulated vitamin D3 influence salivary gland structure and function under calcium-deficient conditions?

## Materials and methods

The current study’s manuscript complies with the reporting requirements for animal research set forth by ARRIVE guidelines [24].

Vitamin D3 (111000 IU D3) was obtained from Pharaonia Pharmaceuticals (Alexandria, Egypt). Gattefossé S.A., Saint-Priest, France, generously donated Labrafac^®^ (lipophile WL 1349, caprylic-capric acid triglycerides, European Pharmacopoeia, IVth, 2002). BASF (Ludwigshafen, Germany) supplied Kolliphor^®^ HS 15, also called Solutol^®^ HS 15, which is a mixture of free polyethylene glycol 660 and polyethylene glycol 660 hydroxystearate that complies with the European Pharmacopoeia IV edition (2002). Lipoid^®^ S100, a soybean lecithin containing 69% phosphatidylcholine, was generously provided by Lipoïd GmbH (Ludwigshafen, Germany). All other reagents and solvents used were of analytical grade. Concerning HPLC analysis, Acetonitrile and Isopropanol used are of HPLC grade and Tetrahydrofuran is of analytical grade.

### Animal experimentation

#### Animals and ethical statement

This study received approval from the Research Ethics Committee (Approval No. 2024/221) at the Faculty of Dentistry, AASTMT Alamein campus. Thirty-six healthy adult male albino rats, weighing between 150 and 165 g and approximately six months old, were obtained from the Medical Research Institute of the University of Alexanderia [25]. They were kept in controlled laboratory settings. The cages were cleaned twice a day [24].

#### Calculating the sample size

Sample size was calculated using G Power version 3.1.9.2 and Power Analysis and Sample Size Software (PASS 2020), “NCSS, LLC. Kaysville, Utah, USA, ncss.com/software/pass”. Al-Serwi et al. (2021) showed that the average number of submandibular serous acini cells was significantly higher (*p* < 0.05) in the group treated with nano vitamin D (35 ± 4.3) than in the group treated with conventional vitamin D (28 ± 3.1) [14]. Thus, a minimal total hypothesized sample size of 36 eligible rats (12 per group) is needed to evaluate the protective potential of vitamin D3-loaded lipid nano capsules (LNCs) versus conventional vitamin D3 in countering fatty degeneration in parotid salivary glands caused by a calcium-deficient diet; taking into consideration 95% confidence level, effect size of 20% (difference in the weight gain) and 80% power using *Chi-square* test [26].

$${\rm{Sample}}\:{\rm{size}}\:{\rm{ = }}\:\frac{{\frac{{{z^2}p(1 - p)}}{{{e^2}}}}}{{1 + \left( {\frac{{{z^2}p\left( {1 - p} \right)}}{{{e^2}N}}} \right)}}$$

The animals were split up into three equal groups using a random number generator:

- **Group I (Calcium-deficient group**,** n = 12)**: Rats in this group were fed a diet low in calcium, containing only 0.1–0.3% calcium [27].
- **Group II (Calcium-deficient diet group + conventional vitamin D3**,** n = 12)**: These rats were provided with a calcium-deficient diet al.ong with a daily oral dose of conventional vitamin D3 (Vitamin D3 oral drops) Vidrop^®^ Medical Union Pharmaceuticals (MUP)/ Egypt” (40IU/Kg of body weight orally daily) by gavage.
- **Group III (Calcium-deficient diet + Vitamin D3-loaded-LNC**,** n = 12)**: This group received a calcium-deficient diet al.ong with a daily oral dose of vitamin D3-loaded LNC at 40 IU/kg of body weight orally by gavage [14].

### Preparation of blank and vitamin D3-loaded lipid nano-capsules

Blank lipid nano capsules (Blank-LNCs) were formulated using the phase inversion temperature (PIT) method followed by dilution, according to previously established protocols [28]. Kolliphor^®^ HS 15, Labrafac^®^, and deionized water were combined in a weight ratio of 1:1:3 using magnetic stirring. Sodium chloride (0.44% w/v) and Lipoid^®^ (0.75% w/v) were then added to the oil/water/surfactant mixture. The resulting formulation underwent three heating and cooling cycles between 65 °C and 85 °C. During the final cycle, the mixture was rapidly cooled (quenched) at the phase inversion temperature (79 °C) by adding cold deionized water (0–2 °C) at a 3.5-fold dilution. The dispersion was then gently stirred for 5 min using a magnetic stirrer, filtered through a 0.45 μm Millipore syringe filter, and stored at 4 °C for subsequent analysis [28, 29]. For the preparation of vitamin D3-loaded LNCs, vitamin D3 (4 mg/mL) was directly mixed with the other ingredients from the outset, and the process was followed as described for the blank LNCs.

### Physicochemical characterization of the lipid nano-capsules (LNCs)

#### Determination of particle size and zeta potential

At 25 °C and a fixed angle of 173°, the z-average particle size and polydispersity index (PDI) were measured using dynamic light scattering (DLS) with a Malvern Zetasizer^®^. A 4 mW He-Ne laser with a wavelength of 633 nm was used for the measurements (Zetasizer^®^ Nano ZS series DTS 1060, Malvern Instruments S.A., Worcestershire, UK). The zeta potential was measured at 25 °C in water (dielectric constant 79, refractive index 1.32, viscosity 0.88 cP) with a cell voltage of 150 V and a current of 5 mA. Before testing, samples were diluted at a ratio of 1:20 v/v with filtered deionized water. All measurements were performed three times [30].

#### Microscopic morphology of loaded and blank lipid nano capsules

Vitamin D3-loaded LNCs (4 mg/mL) were examined using transmission electron microscopy (TEM, JEM-100 CX, JEOL, Japan). A 2% w/v uranyl acetate solution was used to stain the LNC dispersions after diluting them with deionized water to a 1:60 v/v ratio and sprayed onto copper grids. Samples were allowed to dry at room temperature before being photographed at 80 kV and 50,000x magnification [31].

#### Investigation of interaction between vitamin D3 and LNC components using fourier-transform infrared spectroscopy (FT-IR)

The interaction between Vitamin D3 and the components of LNCs was studied using Fourier Transform Infrared (FTIR) spectroscopy (IRTracer-100, SNO: A21705901755, Shimadzu, Japan). Aerosol-dried formulations (4:1 ratio) were blended with potassium bromide, then compressed into pellets for analysis. The spectral analysis was conducted over a wavelength range of 4000 to 500 cm¹ with a resolution of 4 cm¹, averaging 20 scans [32].

#### Determination of vitamin D3 level by HPLC

The quantification of Vitamin D3 in Vitamin D₃-loaded lipid nano capsules (LNCs) was performed using a modified, stability-indicating high-performance liquid chromatography (HPLC) method [33]. An aliquot of 200 µL of the LNC formulation was diluted with tetrahydrofuran (THF) in a 1:1 ratio, and the volume was adjusted to 10 mL using isopropanol in a volumetric flask. The resulting solution was filtered through a 0.45 μm polytetrafluoroethylene (PTFE) syringe filter before analysis.

Chromatographic separation was achieved using an Agilent C18 column (5 μm, 4.6 × 150 mm) maintained at 55 ± 1 °C. The mobile phase consisted of acetonitrile and HPLC-grade water in a 99:1 (v/v) ratio, delivered at a 1.5 mL/min flow rate. The injection volume was 100 µL, and the total run time was 5.2 min. Detection was carried out at a wavelength of 265 nm for selective monitoring of Vitamin D3. The analysis was conducted using an Agilent 1260 Infinity II.

An amount of 100 mg of standard Vitamin D3 (100,000 IU/g) was weighed and dissolved in a 50 mL volumetric flask with a few drops of water. Sonicated for 5 min. Volume completed with isopropanol.2 mL of the filtered prepared stock solution was diluted to 50 mL with isopropanol. Sample filtered using a PTFE syringe filter membrane (0.45 μm).

##### Test preparation

Transferred 200 µL of the sample and 200 µL of tetrahydrofuran (THF) into a 10 mL volumetric flask. Volume completed with isopropanol and mixed well. Sample filtered using a PTFE syringe filter membrane (0.45 μm).

##### Placebo preparation

200 µL of THF was transferred into a 10 mL volumetric flask. Volume was finished using isopropanol. A PTFE syringe filter membrane (0.45 μm) was used to filter the sample.

#### Entrapment efficiency [33]

The ultrafiltration/ultracentrifugation method was used to measure the entrapment efficiency (EE%). The LNCs dispersion was centrifuged at 3663 × g for 30 min at 4 °C in an ultra-centrifugal concentrator (Sartorius™ Vivaspin6™, MWCO 100,000). To ascertain the drug content, HPLC was used to quantify the amount of unentrapped vitamin D3 in the filtrate. The following formula was then used to determine the vitamin D3 concentration based on a standard vitamin D3 concentration:

$$ \begin{array}{l}{\bf{Total}}\,{\bf{drug}}\,\left( {{\bf{mg}}} \right) - {\bf{unentrapped}}\,{\bf{drug}}\,\left( {{\bf{mg}}} \right)/\\\left( {{\bf{Total}}\,{\bf{drug}}\,\left( {{\bf{mg}}} \right)} \right)\, \times \,{\bf{100}}\,{\bf{is}}\,{\bf{the}}\,{\bf{EE}}\% \end{array} $$

### Outcome measures

#### Weight of the body [34]

Throughout the 8-week trial, the body weight of every rat in all groups was recorded once a week.

#### Biochemical analysis [35]

Blood samples were taken from the rats’ tail veins. Centrifugation (3000 rpm for 15 min) was used to extract the serum, which was then kept at -80 °C. Rat-specific insulin ELISA kits were used to measure blood insulin levels. Serum glucose and calcium concentrations were determined using an auto-analyzer. At the end of the experiment, animals were euthanized via intraperitoneal injection of sodium thiopental with a lethal dose (120 mg/kg, overdose; Nembutal, Akron, IL, USA) [36]. After that, the biological tissue samples were collected.

#### The histological process [34]

The parotid salivary glands were cleaned, dehydrated, and fixed in 10% neutral-buffered formalin. Following increasing ethanol concentrations, they were implanted in paraffin wax after being washed with xylene. Hematoxylin and Eosin-stained thin slices (4 μm) were produced and viewed under a light microscope.

#### Ultrastructural specimen preparations [37]

The samples were promptly preserved for two hours in 2.5% glutaraldehyde buffered with 0.1 mol/L PBS (pH 7.4). After that, they were postfixed for one to four hours at 4 °C in 1% osmium tetroxide in the same buffer. Following fixation, the samples were prepared, placed within BEEM capsules with epoxy glue, and incubated for 24 h at 60 °C. A glass knife was used in an ultramicrotome to cut extremely thin slices that ranged in thickness from 50 to 100 nm. These slices were stained with uranyl acetate and lead citrate.

#### Calculating the quantity of immature secretory granules [38]

Sections of the parotid salivary gland were examined at ×2500 magnification using a transmission electron microscope (Tokyo, Japan). The areas of immature secretory granules and acinar cells were examined using Image Pro Plus v6.0 for 8-bit pictures. TEM images of the serous acini’s apical parts were chosen randomly. TEM images of the apical parts of the serous acini were selected using simple random sampling. A numbered grid was applied to each section, and fields were randomly chosen using a random number generator to ensure unbiased and representative image selection.

The area fraction of immature secretory granule cores, Fim, was calculated as follows:

A cell (n) is the area of the cell in slice n of the image, while Aim(n) is the total of the areas of all the immature secretory granules in slice n.

$$\:{f}_{im}=\sum_{n=1}^{N}\frac{{A}_{im\:}\left(n\right)}{{A}_{cell}\left(n\right)}$$

#### Statistical evaluation and data handling [39]

The data was analyzed using IBM SPSS for Windows (version 23.0). A comprehensive review of the data was carried out to detect and correct any errors during data entry. Normality was assessed using the Shapiro-Wilk test, and all variables followed a normal distribution. As a result, means and standard deviations (SDs) were computed. One-way ANOVA and Tukey’s HSD (Honestly Significant Difference) test were used to analyze differences in body weight, food intake, and biochemical parameters across the three groups. A criterion of *P* < 0.05 was used to evaluate statistical significance.

## Results

### Physicochemical characterization of LNCs

#### Determination of particle size and zeta potential

The particle size and distribution (polydispersity index) are essential factors that reflect the uniformity of the nano dispersions produced. This study looked at how the average particle size of LNCs was affected by the percentages of fat and vitamin D3. Table 1 illustrates that the mean particle size distribution of the manufactured LNCs was 50.5 nm for the blank LNC and 38.8 nm for the LNC loaded with vitamin D3.

The electric charge at the particle surface is described by the zeta potential (ZP), which also sheds light on the forces that repel other particles or droplets. It influences the release kinetics and biological destiny of nanoparticles and is an essential indication for forecasting the physical stability of colloidal systems.

As shown in Table 1, Blank and Vitamin D3 loaded LNC formulations exhibited weak to moderate negative surface charges (ranging from − 7.76 to -14.3 mV).

Vitamin D3-loaded and the blank LNC formulations prepared in this study had PDI values of 0.125 and 0.157, respectively.

**Table 1:** Colloidal properties of blank lipid nano capsules and vitamin D3-loaded nano capsules

LNC	Particle size, nm	PDI	Zeta potential (ζ), mv
Blank LNC	50.5	0.125	-7.76
Vitamin D3-Loaded LNC	38.82	0.157	-14.3

#### Microscopic morphology of loaded and blank lipid nano capsules

**Fig. 1:**
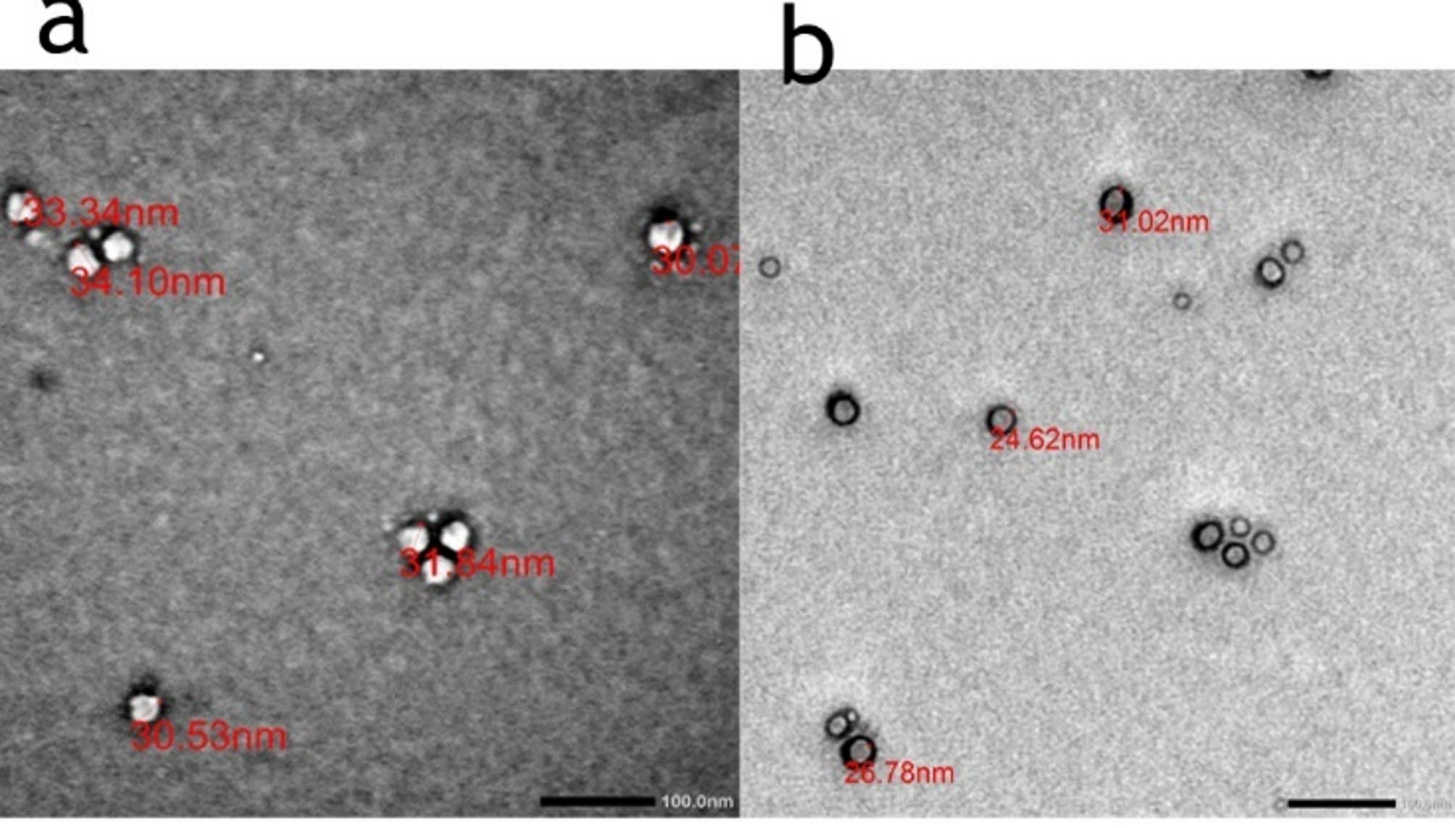
TEM images of (**a**) Blank LNC and (**b**) vitamin D3 loaded LNC

TEM images of Blank LNCs, VitaminD3 loaded LNCs showed a spherical morphology, which was homogenously distributed as shown in Fig. 1.

#### Investigation of interaction between vitamin D3 and LNC components using fourier-transform infrared spectroscopy (FT-IR)

**Fig. 2:**
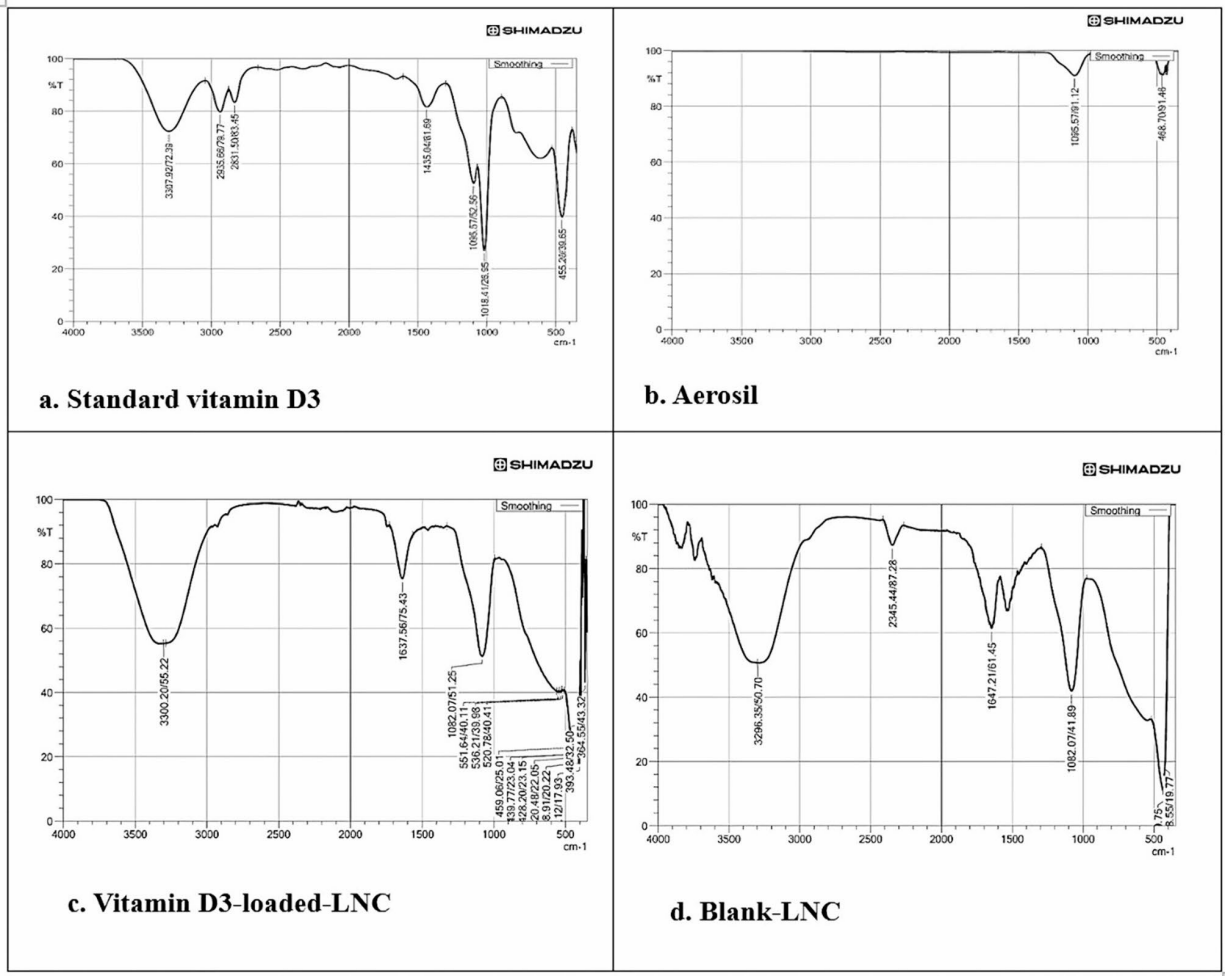
FTIR spectra of vitamin D_3_ (**a**), standard Vitamin D3 (**b**), Aerosol (**c**), Vitamin D3-loaded-LNC (**d**), Blank-LNC

Similar bands that are characteristic of vitamin D3 are seen in the IR spectrum of the formulation containing vitamin D3 at 3315 cm − 1 (hydrogen bond -OH stretching), 2943 cm − 1 (alkyl C–H stretches), 2352 cm − 1 (C–O group), and 1097.5 cm − 1 (C–H bends). In contrast, the blank LNCs displayed a C-O band at 1110 cm − 1, aliphatic C-H bonds at 2866 and 2925 cm − 1, ester groups at 1743.33 cm − 1, and an O-H stretch band at 3464.49 cm − 1.

All the distinguishing bands present in the blank LNCs, as well as the characteristic peaks of the medication, were visible in the IR spectrum of vitamin D3-loaded LNCs, with only a small shift. Figure 2.

#### Determination of vitamin D3 level by HPLC and entrapment efficiency

The HPLC method developed for the analysis of vitamin D3 in lipid nano capsules demonstrated high precision and accuracy. The retention times for vitamin D3 in all samples were consistent, with lipid nano capsules showing a slightly delayed peak due to the encapsulation matrix. The placebo samples confirmed the absence of vitamin D3, validating the method’s specificity. The content of vitamin D3 evaluated quantitatively denoted a percentage recovery value of 105.11. % EE showed 99.8% based on the content calculations.

### Changes in body weight

**Fig. 3:**
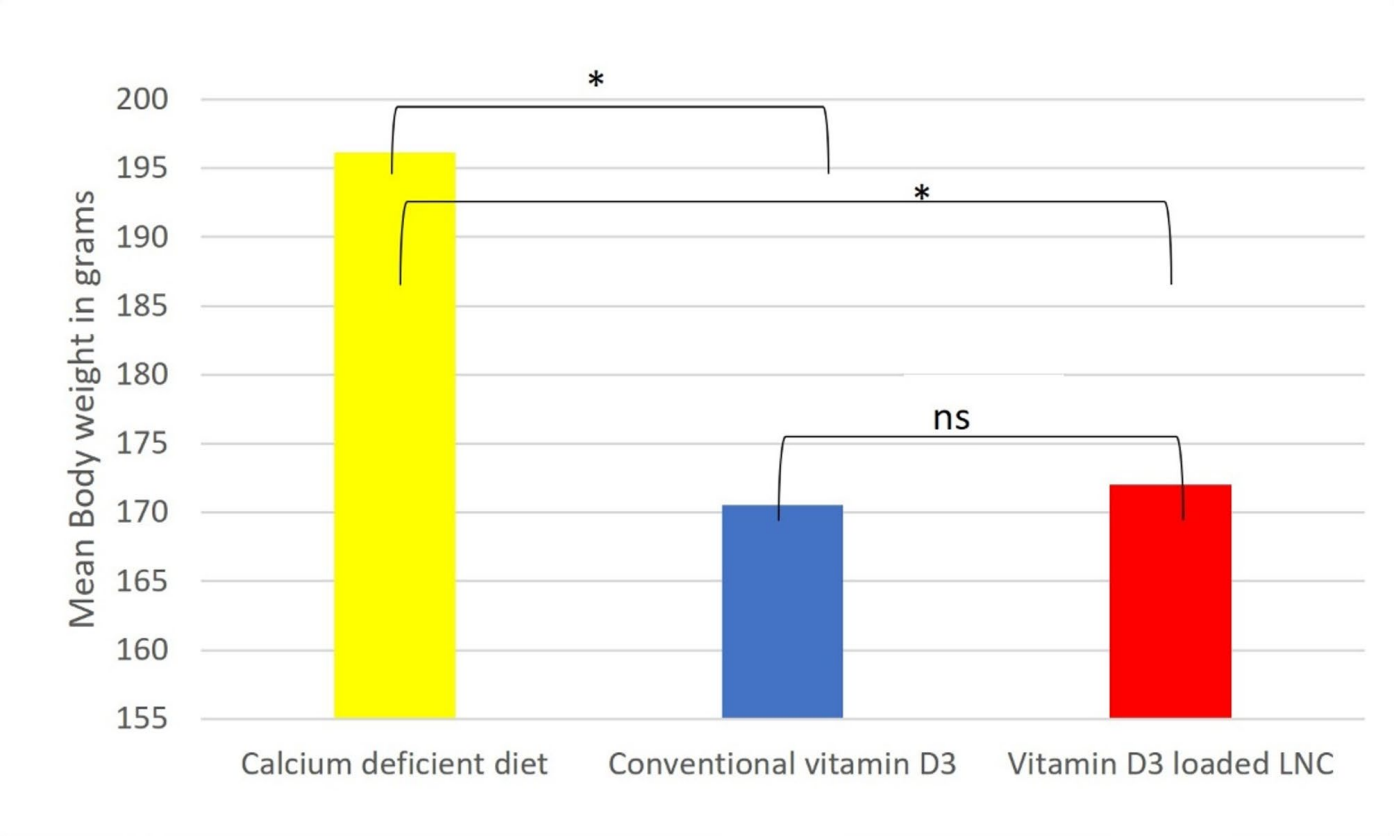
Image illustration between the three groups: low calcium diet, conventional vitamin D3 group, and Vitamin D3-loaded-LNC, showing the differences in the mean body weight. (* Statistically significant, ns: non-statistically significant, and a significance level of 95% (α = 0.05)

The means ± SD weight gain during the experiment among the calcium-deficient diet, conventional vitamin D3, and Vitamin D3-loaded-LNC groups were (196.1875 ± 19.6, 170.5625 ± 18.5, 172.0375 ± 15), respectively, and was statistically significant between group II and group I (*p* = 0.016058), and between group III and I (*p* = 0.01784). However, there were no significant differences between the conventional vitamin D3 and Vitamin D3-loaded-LNC groups (*p* = 0.864825). Figure 3. Using Tukey’s honestly significant difference analysis and one-way ANOVA, all the data were gathered and statistically examined.

### Serological analysis

#### Calcium serum level

**Fig. 4:**
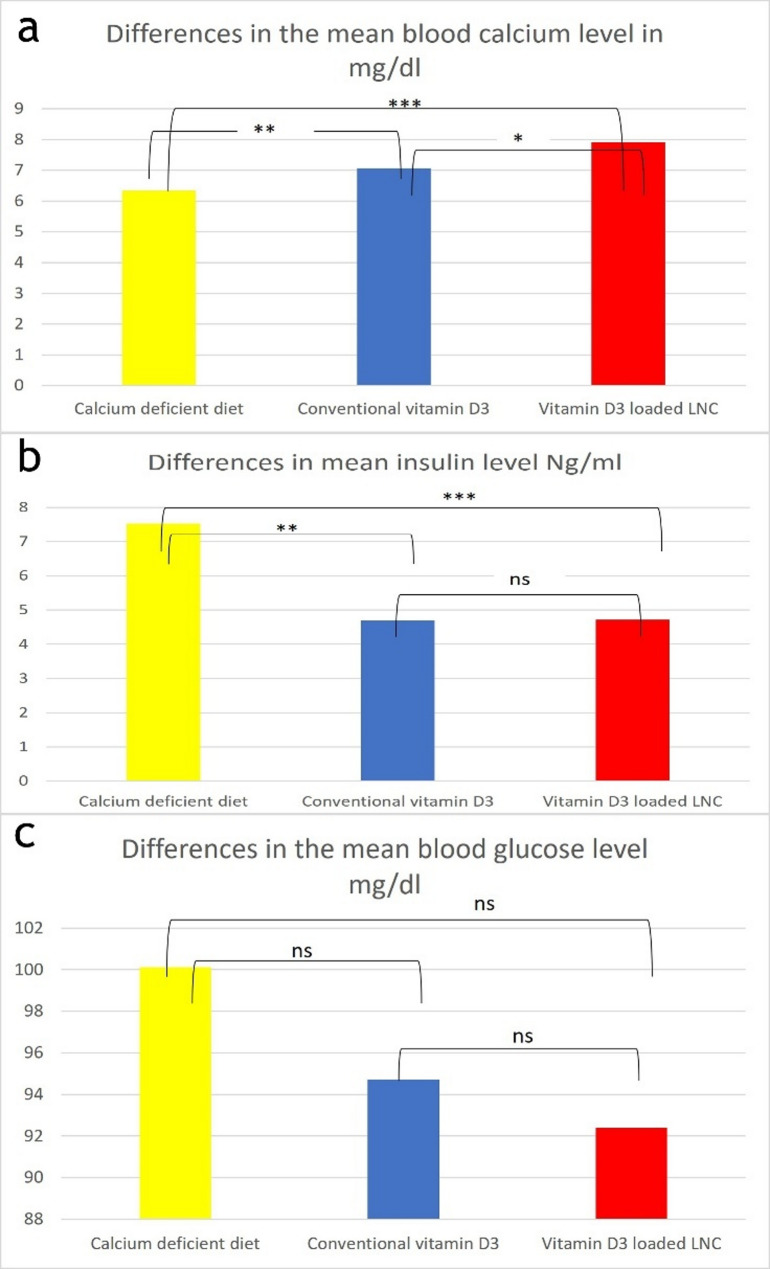
Image illustration between the three groups: low calcium diet, conventional vitamin D3 group, and vitamin D3-loaded-LNC group, showing the mean differences between them in the **a**) calcium level (mg/dl), **b**)insulin level(ng/ml), and **c**)glucose blood serum level(mg/dl). (*for *p* < 0.05, ** for *p* < 0.01, ***for *p* < 0.001, ns = nonsignificant)

The means ± SD of calcium serum level among the calcium-deficient diet, conventional vitamin D3, and Vitamin D3-loaded-LNC groups were (6.35 ± 0.39,7.07 ± 0.5, 7.92 ± 0.9), respectively, and there were statistically significant differences between the three groups. There was a significant difference between the conventional vitamin D3 and the Vitamin D3-loaded-LNC groups (*p* = 0.0212) and was statistically significant between group II and group I (*p* = 0.00322), and between group III and I (*p* < 0.001) with the Vitamin D3-loaded-LNC group exhibiting the highest and the calcium-deficient group exhibiting the lowest calcium levels. Figure 4a. Using Tukey’s honestly significant difference analysis and one-way ANOVA, all the data were gathered and statistically examined.

#### Insulin in blood level

The means ± SD of blood insulin levels among the calcium-deficient diet, conventional vitamin D3, and Vitamin D3-loaded-LNC groups were (7.5 ± 0.9,4.7 ± 0.39,4.72 ± 0.3) and were statistically significant between group II and group I (*p* < 0.001), and between group III and I (*p* < 0.001). However, there were no significant differences between the conventional vitamin D3 and Vitamin D3-loaded-LNC groups (*p* = 0.903). Figure 4b. Using Tukey’s honestly significant difference analysis and one-way ANOVA, all the data were gathered and statistically examined.

#### Blood glucose level

The means ± SD of blood glucose levels among the calcium-deficient diet, conventional vitamin D3, and Vitamin D3-loaded-LNC groups were (100 ± 6.88,93.7 ± 5.8,88.6 ± 10.6), and there was no statistically significant difference between group II and group I (*p* = 0.077), and between group III and I (*p* = 0.136). Moreover, there were no significant differences between the conventional vitamin D3 and Vitamin D3-loaded-LNC groups (*p* = 0.638). Figure 4c. Using Tukey’s honestly significant difference analysis and one-way ANOVA, all the data were gathered and statistically examined.

### Determination of the number of immature secretory granules per acinar cell from electron micrograph images

**Fig. 5:**
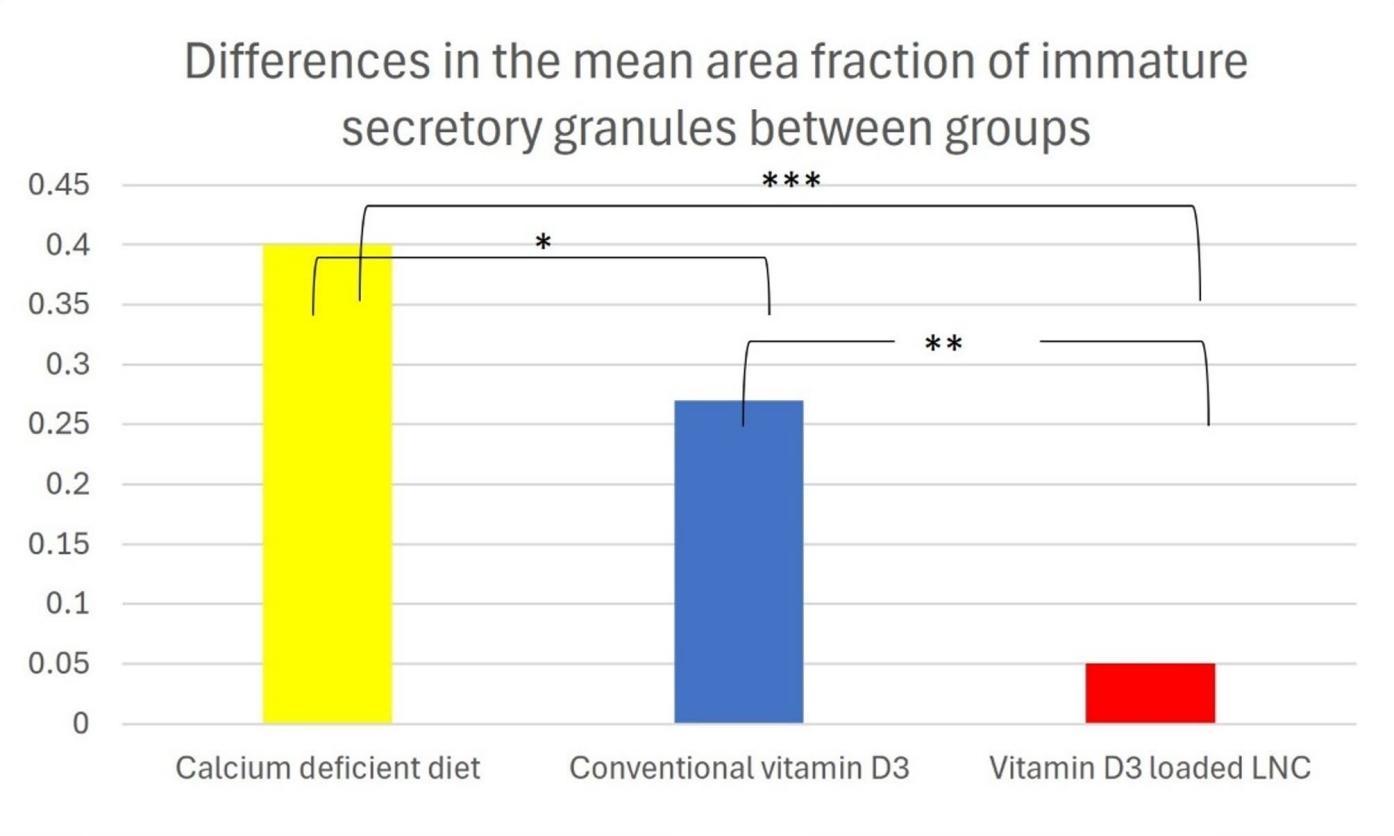
Bar chart between the three relative groups: low calcium diet, conventional vitamin D3 group, and Vitamin D3-loaded-LNC group regarding the area fraction of immature secretory granules. * For *p* < 0.05, ** for *p* < 0.01, *** for *p* < 0.001

The mean ± SD of the area fraction of immature secretory granules (Fim) for the calcium-deficient diet, conventional vitamin D3, and Vitamin D3-loaded-LNC groups were 0.4 ± 0.14, 0.27 ± 0.068, and 0.05 ± 0.02, respectively. Significant differences were observed between the conventional vitamin D3 and calcium-deficient diet group (*p* = 0.016) as well as between the calcium-deficient diet and the Vitamin D3-loaded-LNC group (*p* < 0.001). Furthermore, there were significant differences between the conventional vitamin D3 and the Vitamin D3-loaded-LNC groups (*p* < 0.01), with the Vitamin D3-loaded-LNC group showing the lowest area fraction of immature secretory granules. Figure 5. Using Tukey’s honestly significant difference analysis and one-way ANOVA, all the data were gathered and statistically examined.

Area fraction of immature secretory granules:

$$\:\frac{\varvec{A}\varvec{i}\varvec{m}\:\left(\varvec{n}\right)}{\varvec{A}\varvec{c}\varvec{e}\varvec{l}\varvec{l}\:\left(\varvec{n}\right)}$$

### Histological results

**Fig. 6:**
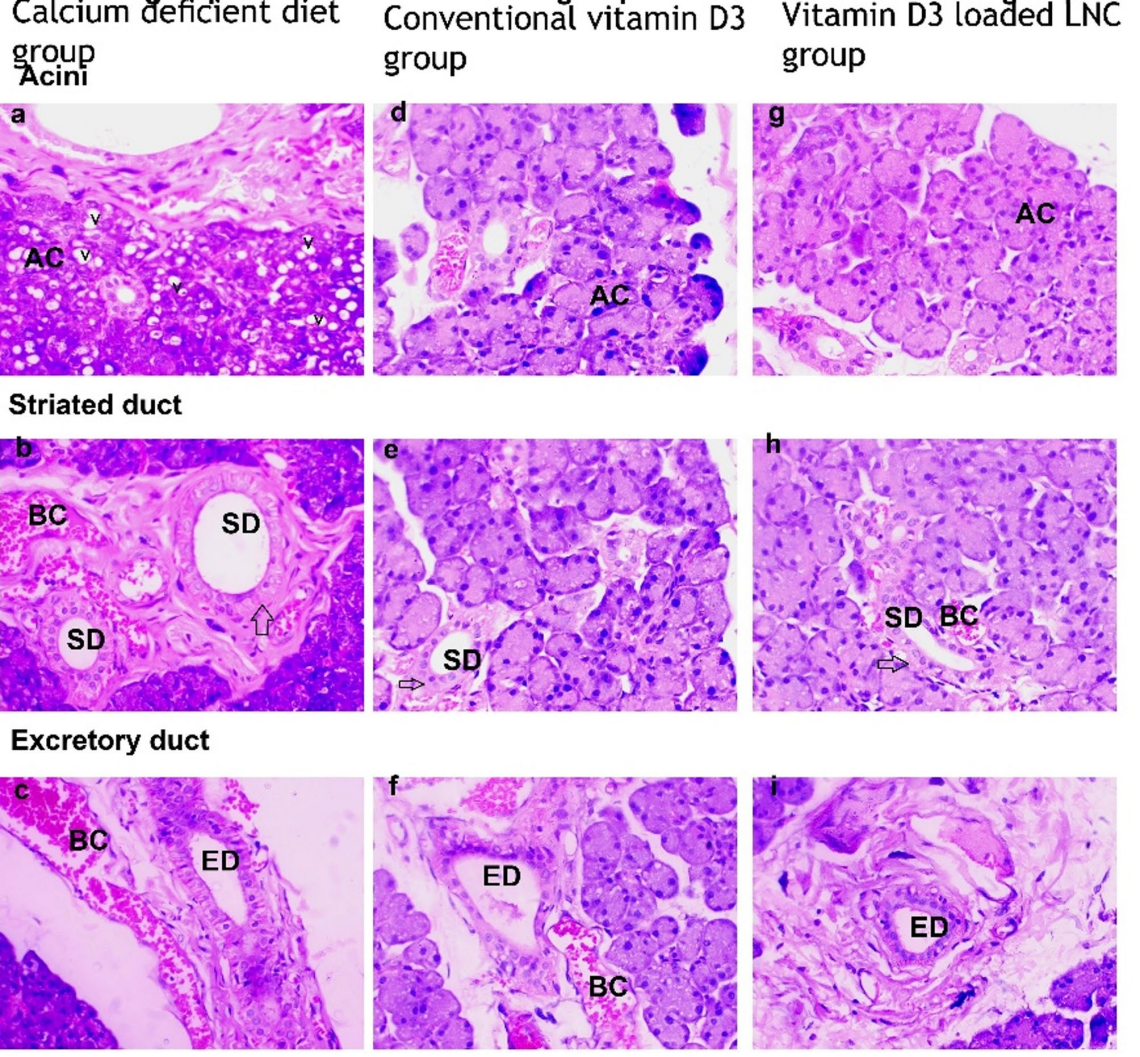
Light micrograph (LM) of the three groups showing In the calcium-deficient diet group, the acini (AC) displayed atrophy and an expanded lumen in the striated duct (SD), along with a loss of striation (arrow) The excretory duct (ED) showed abnormal lumen expansion and dilated blood capillaries (BC). Additionally, the epithelial lining had deteriorated [H&E Stain, original magnification × 400] In the conventional Vitamin D3 group, the serous acini (AC) are moderately preserved the striated duct (SD) is well-preserved, surrounded by basal striations (arrow). The excretory duct (ED) is slightly preserved, with a slightly expanded lumen encircled by mild connective tissue fibrosis. [H&E: original magnification ×400] In the Vitamin D3-loaded-LNC group, the parotid salivary gland exhibits well-preserved normal histological structure, with serous acini (AC). The striated duct (SD) is also well-preserved with basal striations (arrow The typical excretory duct (ED) is encircled by connective tissue fibers and cells, [H&E: original magnification ×400]

- **Light microscopic results**, Fig. 6.

- **Group I (Calcium-deficient diet group)**.

The acini of these individuals exhibited degeneration, with many cytoplasmic vacuoles and pyknotic nuclei (Fig. 6a). The secretory striated duct showed lumen expansion, loss of its striations, dilated blood capillaries filled, and disintegration of the endothelial layer of the duct (Fig. 6b). The excretory duct displayed abnormal lumen widening, along with disintegration of the endothelial layer and fibrosis in the connective tissue (Fig. 6c).

- **Group II (Conventional Vitamin D3 group)**.

Moderately preserved serous acini, with few separations between the acini (Fig. 6d). Well-preserved striated duct surrounded by basal striations and epithelial columns, near a dilated blood capillary with RBCs (Fig. 6e). Slightly preserved excretory duct with a relatively dilated lumen that is lined by pseudostratified columnar epithelium, surrounded by some fibrosis. Note the dilated blood capillaries associated with the duct (Fig. 6f).

- **Group III** Vitamin D3-loaded-LNC.

Histological analysis of the control group images (Fig. 6) revealed normal acini, typical cytoplasm, and standard stainability (Fig. 6g). The secretory striated ducts exhibited clear basal striations (Fig. 6h). A normal excretory duct encircling a broad lumen and bordered with pseudostratified epithelium covered in fibrosis, and contains some stuck saliva (Fig. 6i).

**Fig. 7:**
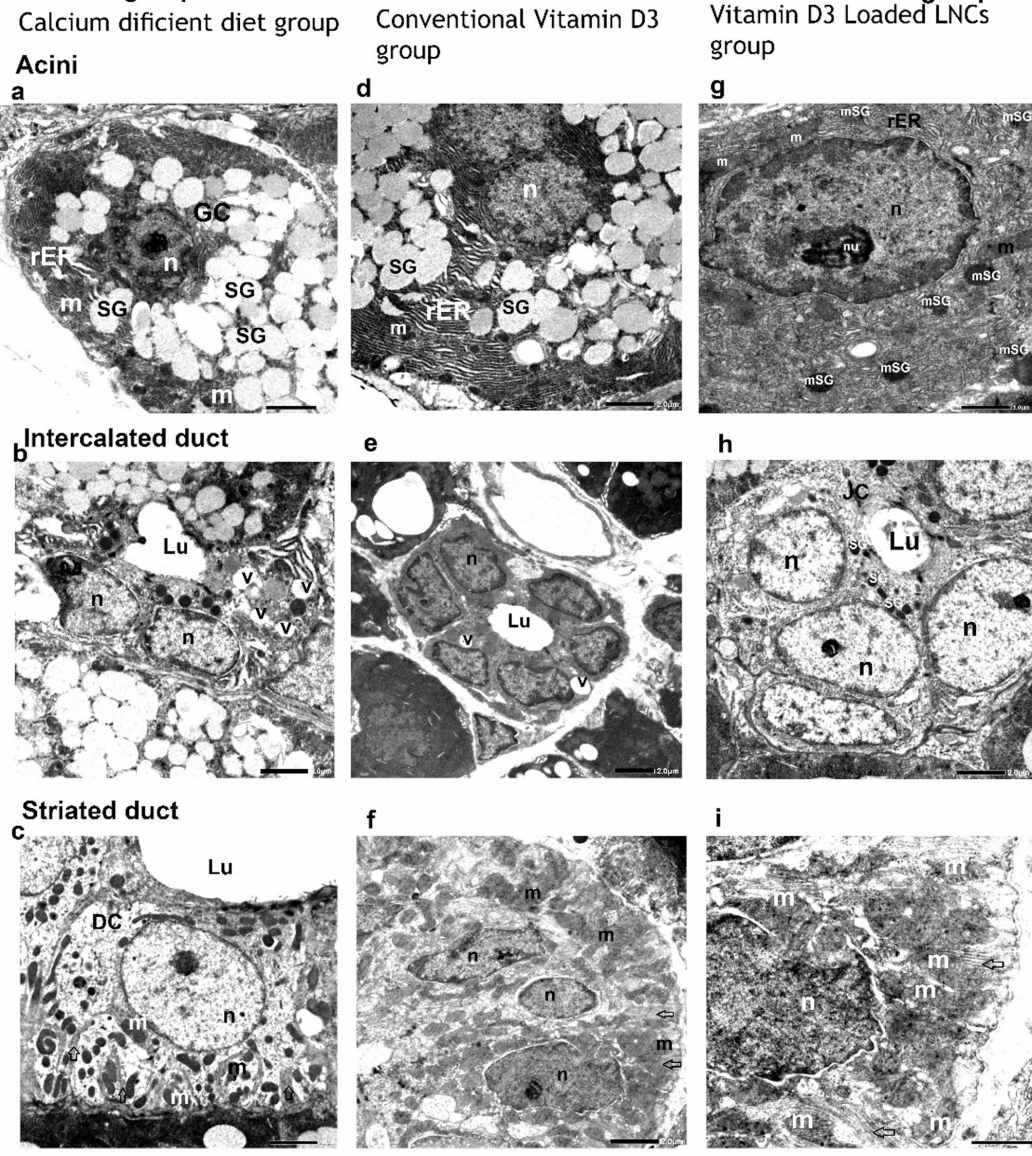
TEM of the parotid gland of the three different groups showing: **In the calcium-deficient diet group**, in addition to damaged mitochondria (m), and several immature secretory granules (SG). The serous cell displayed a heterochromatic, deteriorated nucleus (n), several enlarged rough endoplasmic reticula (rER), and a Golgi complex (GC). Notable spaces between adjacent cells were observed (original magnification ×2500). An abnormal intercalated duct, a slightly widenedlumen (Lu), and disorganized, degenerated organelles (original magnification ×2500). The secretory striated duct displayed significant lumen widening (Lu), loss of basal infoldings, deteriorated mitochondria (m), and disintegrated cytoplasm (DC) (original magnification ×2500) **In the conventional vitamin D3 group**, the serous cell’s cytoplasm contains a large, fairly organized rough endoplasmic reticulum (rER), a few normal mitochondria(m), and a normal nucleus (n). Numerous immature electron-lucent secretory granules (SG) were observed (original magnification ×5000). normal intercalated duct, surrounded by a narrow lumen (Lu) with a few vacuoles (v) in the cytoplasm (original magnification ×2500). A normal part of the secretory striated duct that has euchromatic nuclei that are normal (n), partially preserved basal infoldings (arrows), (original magnification ×6000) **In the Vitamin D3-loaded-LNC group**: A portion of a serous acinus showed a typical nuclear membrane and a normal euchromatic nucleus (n) and prominent nucleolus (nu), abundant normal mitochondria (m), and a well-organized rough endoplasmic reticulum (rER) in the serous cell’s cytoplasm. A few mature electron-dense secretory granules (mSG) were present (original magnification ×5000). A typical intercalated duct (original magnification ×2500). The secretory striated duct with well-preserved basal infoldings (arrows), original magnification× 6000) Note: JC: junctional complex

b) **Electron microscopic results**: Fig. 7.

- **Group I (Calcium-deficient diet group)**.

Examination of the various organelles and components found in the parotid salivary gland revealed a serous cell with a heterochromatic, degraded nucleus, enlarged Golgi complex, dilated rough endoplasmic reticula, uneven nuclear membrane, and deteriorated mitochondria. Distancing between adjacent cells was observed, along with numerous immature secretory granules (Fig. 7a). Additionally, an abnormal intercalated duct was seen, with partial deterioration of the epithelial lining and slight lumen widening, as well as vacuolations in the duct’s cytoplasm (Fig. 7b). A big, broad lumen with dispersed deteriorated mitochondria and the absence of baseline folds was seen in the secretory striated duct. (Fig. 7c)

- **Group II (Conventional Vitamin D3 group)**.

The transmission electron microscope analysis revealed a section of a serous acinus displaying typical nuclear membranes and normal euchromatic nuclei, abundant rough endoplasmic reticulum in the serous cell’s cytoplasm that is moderately organized, and a few normal mitochondria, along with numerous immatures, electron-lucent secretory granules (Fig. 7d). A partially preserved intercalated duct composed of a small channel surrounded by cubic cells with rounded, centrally positioned euchromatic nuclei, with few vacuoles were present in the cytoplasm (Fig. 7e). High columnar cells with typical euchromatic nuclei bordered the striated duct and partially preserved basal infoldings, containing a large number of mitochondria oriented longitudinally (Fig. 7f).

- **Group III** Vitamin D3-loaded-LNC.

This group revealed a portion of a serous cell with a regular nuclear membrane, a typical euchromatic nucleus, and a large quantity of neatly arranged rough endoplasmic reticulum, along with normal mitochondria. Additionally, mature secretory granules were observed (Fig. 7g). The intercalated duct appeared normal, consisting of cuboidal cells having little electron-dense secretory granules and a rounded, centrally placed euchromatic nucleus with a small lumen. The duct also exhibited a well-formed junction complex (Fig. 7h). Long columnar cells lined the regular secretory striated duct, which also showed well-formed basal folds and normal euchromatic nuclei, housing a significant number of mitochondria oriented longitudinally (Fig. 7f).

## Discussion

Calcium is necessary for blood coagulation, muscle contraction, and healthy bones and teeth, heart rhythm regulation, and nerve function. About 99% of the body’s calcium is stored in bones, while the rest is found in the blood, muscles, and other tissues [40, 41].

An insufficient calcium intake in the diet causes hypocalcemia, resulting in osteoporosis [42]. Hypocalcemia can cause muscle cramps, tingling, and spasms, as well as seizures and abnormal heart rhythms [43, 44]. Furthermore, hypocalcemia can promote lipogenesis while decreasing lipolysis, leading to obesity [45]. However, it can impair overall health by causing weakening in the hair and nails, and physical inactivity [46]. Additionally, it might impair dental health by decreasing salivary gland activity and secretions [6].

Previous studies examined the effect of vitamin C and E on the salivary gland under many conditions. Both Shamel et al. (2024) [47] and Ibuki et al. (2020) [48] investigated the protective effects of vitamin C on salivary gland health under stress conditions. Shamel et al. demonstrated that vitamin C effectively reduced submandibular gland toxicity caused by copper oxide nanoparticles in rats, preserving tissue structure and lowering markers of cell damage. Similarly, Ibuki et al. found that vitamin C supplementation improved the antioxidant defense in the submandibular glands of diabetic rats, while vitamin E unexpectedly increased oxidative stress. Together, these studies highlight the beneficial role of vitamin C in mitigating oxidative and toxic damage in salivary glands.

Moreover, Toan NK et al. (2024) [49] conducted a study to evaluate the potential of ascorbic acid in treating salivary gland dysfunction in an advanced aging mouse model. The findings revealed that ascorbic acid treatment enhanced the expression of acetylcholine receptors by activating the DNA demethylation enzyme TET2. This suggests that ascorbic acid may help restore salivary gland function by compensating for deficiencies in its natural biosynthesis.

Bakr MM et al. (2021) demonstrated that vitamin E possesses strong antioxidant capabilities, effectively counteracting the cytotoxic effects induced by silver nanoparticles (AgNPs) [50].

Vitamin D was chosen over other vitamins due to its multifaceted role in maintaining glandular health, modulating immune responses, and regulating calcium and phosphate balance—all of which are critical for the proper functioning of the salivary glands. Unlike antioxidants such as vitamins C and E, which primarily combat oxidative stress, vitamin D has been shown to influence gene expression related to cell differentiation, inflammation, and immune regulation. Additionally, vitamin D receptors are expressed in salivary gland tissues, indicating a direct biological pathway through which vitamin D can exert its effects. Vitamin D deficiency is associated with increased risk of insulin resistance and type 2 diabetes, as it impairs insulin secretion, reduces insulin sensitivity, and promotes inflammation and oxidative stress, all of which disrupt glucose metabolism [51]. Its widespread deficiency in the general population further supports the need to investigate its therapeutic potential in salivary gland disorders [52, 53].

Nanotechnology has revolutionized modern dentistry by offering innovative solutions to various dental issues. Nanoparticles (NPs) possess unique physicochemical and biological properties, such as biocompatibility, high surface area, strength, solubility, chemical reactivity, and antibacterial activity, that enhance their effectiveness. These attributes enable improved diagnosis, prevention, and treatment of several oral conditions [54].

Studies have shown that the bioavailability of encapsulated compounds improves as the particle size decreases, which has been proved with the prepared VitaminD3 loaded LNC in the study exhibited small particle size below 100 nm [29]. Consistent with the Zetasizer’s results, all LNCs had a limited size distribution, were spherical, and were ≤ 100 nm in size. Their physical characteristics were largely consistent with those documented in the literature, particularly about size and shape [55].

Blank and loaded LNC formulations exhibited weak to moderate negative surface charges (ranging from − 7.76 to -14.3 mV), attributed to the negative contribution of phospholipid molecules and the presence of PEG dipoles in their shell [29]. Negative zeta potential can contribute to a more controlled and sustained release of Vitamin D3, improving its bioavailability. LNCs with a negative zeta potential are less likely to be recognized and cleared by the immune system. This prolongs their circulation time, allowing more Vitamin D3 to be absorbed. LNCs with negative zeta potential can protect Vitamin D3 from degradation in the acidic environment of the stomach. This ensures that more of the vitamin reaches the intestines for absorption [22, 56]. The polydispersity index (PDI) measures particle size distribution, ranging from 0 to 1. PDI values between 0.1 and 0.25 indicate a fairly narrow size distribution, while values above 0.5 suggest a broad distribution. This index significantly impacts the physical stability of lipid nano capsules and should ideally be kept below 0.5. The quantities of ingredients, the viscosity of the lipid phase, and the conditions of production are some of the variables that affect the particle size and polydispersity index of LNCs.

The HPLC analysis demonstrated that lipid nano capsules (LNCs) showed a consistent retention time across all samples. This, along with improved thermal stability, highlights LNCs as a superior option for enhancing the efficacy and shelf life of vitamin D3 [57].

In our study, lipid nano capsules (LNCs) offered several advantages over solid lipid nanoparticles (SLNs) and nanostructured lipid carriers (NLCs): LNCs were produced using a solvent-free, low-energy phase inversion temperature method. This method is more energy-efficient and suitable for large-scale production, reducing potential degradation of drug substances. For 10 to 40 min, vitamin D3 remains comparatively stable at 80 and 130 °C. Up to 90% of vitamin D3 is lost at high temperatures of 180 °C and 230 °C. The range of the reaction rate for vitamin D3 degradation was 2.01 to 6.80 × 10^2 sec–1 [58].

Lipid nano capsules provide a more versatile, stable, and efficient delivery system compared to solid lipid nanoparticles and nanostructured lipid carriers, making them suitable for a wider range of applications and administration routes. SLNs and NLCs require high-energy methods like high-pressure homogenization or sonication. LNCs have Core-shell structures with an oily liquid triglyceride core surrounded by a rigid surfactant membrane. This structure allows for versatile formulation strategies, including the encapsulation of both lipophilic and hydrophilic cargoes like NLCs. In addition to improved loading capacity for the cargo. LNCs have prolonged colloid stability in suspension. The rigid membrane helps maintain structural integrity over time. SLNs and NLCs have variable stability; NLCs generally offer better stability than SLNs due to their mixed lipid composition. LNCs are tested for multiple administration routes, including intravenous, subconjunctival, intranasal, oral [59], pulmonary, topical, and intradermal. SLNs and NLCs: are primarily used for oral, topical, and intravenous routes. LNCs are biocompatible, biodegradable, and non-toxic. The mild emulsification settings avoid potential drug substance degradation [60, 61].

To rule out any hormonal changes that might affect the outcomes of a calcium-deficient diet, male rats were employed [62]0.8 weeks was sufficient to induce obesity and raise the oxidative stressors that lead to insulin resistance, sometimes referred to as the prediabetic stage, in rats given a calcium-deficient diet [45].

Because they seem to be more impacted by the deterioration brought on by a diet low in calcium, we selected the parotid glands. Insulin resistance (IR) is caused by oxidative damage to the parotid glands and is associated with their biological anatomy, namely the large number of adipocyte cells in the parotid glands’ parenchyma. ROS are mostly produced in the adipocyte tissue [63].

In contrast to the conventional vitamin D3 and vitamin D3-loaded LNC groups, our study found that a low calcium diet causes a considerable increase in body weight. This was consistent with recent research that found that low dietary calcium consumption increases PTH and 1,25-hydroxy vitamin D levels, which in turn raise intracellular calcium levels in adipocytes, promoting lipogenesis and preventing lipolysis [64]. Vitamin D helps counteract weight gain induced by a calcium-deficient diet by reducing compensatory PTH and 1,25(OH)₂D secretion, thereby lowering intracellular calcium in adipocytes and limiting fat accumulation [65].

The serological results from this investigation demonstrated that the calcium-deficient diet group had considerably lower serum calcium levels than the conventional vitamin D3 and Vitamin D3 loaded LNC groups. In general, the usual diet provides enough calcium. Any changes in the amount of calcium in the food impact the amount in the blood because it is taken from the upper digestive tract to the circulatory system [66]. Vitamin D plays a crucial role in restoring calcium levels by enhancing its intestinal absorption, promoting renal reabsorption, and mobilizing calcium from bone when needed. Furthermore, vitamin D-loaded lipid nanocarriers (LNCs) may improve the bioavailability and stability of vitamin D, leading to more efficient calcium absorption and a more effective restoration of serum calcium levels [14].

According to the study, blood insulin levels significantly increased in rats given a diet low in calcium, suggesting that calcium can enhance insulin sensitivity and pancreatic B cell activity [9]. And was significantly decreased in groups taking conventional vitamin D3 and vitamin D3 loaded lipid nano-capsules, which proves the ability of vitamin D3 to increase insulin sensitivity [14].

The research indicated no significant differences in blood glucose levels among the three groups. However, there was a slight reduction observed in Group II and a more pronounced decrease in Group III. This reduction may be attributed to vitamin D’s role in enhancing insulin sensitivity, which facilitates greater glucose uptake by cells, thereby lowering blood glucose levels [67].

Additionally, histology data from the calcium-deficient diet group revealed some degradation in the excretory duct lining and serous acini cells, as evidenced by the presence of vacuoles in the acini and a reduction in the excretory duct lining. Chronic inflammation brought on by the adipocyte state causes monocytes and neutrophils to release oxygen free radicals through their NADPH oxidase enzyme activity, which is triggered during phagocytosis. The degradation that occurs within the salivary gland is caused by the free radicals [68].

The study found a substantial rise in fibers in connective tissue septa, interlobular ducts, and intralobular ducts due to chronic inflammation, which causes tissue damage and advanced fibrosis. Inflammation and fibrosis are part of the tissue defense, repair, and regeneration cycle [69].

The observed histological features included marked dilation of the salivary ducts, accumulation of saliva, and blood vessel congestion, all of which are indicative of salivary gland dysfunction. According to Garant in his book, these ductal changes may be due to the buildup of secretions resulting from impaired exocytosis. The congestion of blood vessels can be explained by passive hyperemia, a condition where blood accumulates due to inadequate venous outflow from the affected tissue [8].

In contrast, administering both conventional and Vitamin D3-loaded-LNC reduced the structural damage induced by a low-calcium diet. These findings align with earlier studies, supporting the effectiveness and bioavailability of vitamin D in salivary gland function [35, 53, 70].

According to the current study, Vitamin D3-loaded-LNC treatment outperformed conventional vitamin D3 treatment in preventing the serous acini’s degeneration brought on by a low calcium diet. Conventional vitamin D3 moderately preserved the architecture of the serous acini, with few separations between the acini. Well-preserved striated duct with its basal striations. Slightly preserved excretory duct with a relatively dilated lumen surrounded by some connective tissue fibrosis. However, Vitamin D3-loaded-LNC showed significantly better effects, being more efficacious than regular vitamin D. However, Compared to conventional vitamin D3, Vitamin D3-loaded-LNC has superior efficacy. These results were in agreement with a study conducted by Al-Serwi et al., who investigated the effect of vitamin D3 and nano-vitamin D3 on the fatty deterioration in submandibular and sublingual salivary glands [14].

Ultrastructural analysis revealed membrane swelling and degeneration in the rough endoplasmic reticulum(rER) and Golgi apparatus in the group fed a low-calcium diet. This is attributed to the depletion of luminal Ca²^+^ in the rER, which triggers ER stress and activates the unfolded protein response (UPR). Depending on how intense or prolonged the stress is, the UPR may restore normal ER function or lead to cell death. During ER stress, increased calcium is released from the ER into the mitochondria, leading to calcium overload. This accumulation can cause mitochondrial membrane rupture and the release of enzymes that promote apoptosis [71].

Furthermore, the present study revealed that inflammation triggers oxidative stress and diminishes the cell’s antioxidant defenses, resulting in excessive production of free radicals. These radicals interact with lipids and proteins in the membranes of cellular organelles, leading to their breakdown. Additionally, the free radicals can cause DNA mutations and damage, contributing to nuclear degeneration, as demonstrated by our findings [68].

Additionally, calcium has a vital role in the nucleus, where nuclear Ca²⁺ is crucial for managing the transcription factor CREB and its coactivator CBP (CREB-binding protein). Furthermore, nuclear Ca²⁺ has been shown to bind directly to DNA and influence its structure [72].

Also, there was a rise in immature secretory granules, which is in keeping with the results of Garant PR, who proposed that calcium might act as a universal intracellular messenger for a variety of salivary gland receptors, such as acetylcholine and β-adrenergic receptors. In addition to influencing the activity of protein kinases A and C, calcium is essential for promoting exocytosis and secretory granule anchoring and maturation. Secretory proteins condense during the maturity of these granules, a process made possible by the addition of glycosaminoglycans and the creation of calcium bonds to concentrate the proteins [8].

On the opposite side, our results exhibited partial preservation of the cytoplasmic organelles in the conventional vitamin D3 group with partially preserved intercalated and striated ducts, but with few vacuoles in the cytoplasm and a decrease in the number of immature secretory granules. While Vitamin D3-loaded-LNC group illustrated an outstanding preservation of the normal architecture of the cytoplasmic organelles, as well as the duct system as well and a marked reduction in the number of immature secretory granules.

This can be attributed to the role of conventional vitamin D3 in enhancing its function of the glands by improving the transport of metabolites to the acini cells [73], Protecting acinar cells from damage and playing a key role in controlling electrolyte transport between the stroma and the lumen [74]. Vitamin D3 also contributes to the regeneration of intercalated duct cells, as these ducts contain stem cells [75]. Vitamin D3 is recognized for actively promoting calcium-driven serous salivary secretion [76]. In addition, prior research has highlighted the anti-inflammatory properties of vitamin D3 [77, 78].

However, our findings also revealed that Vitamin D3-loaded-LNC outperforms conventional vitamin D3 as an anti-inflammatory agent. Previous studies have shown that the nano-emulsion form of vitamin D3 is significantly more effective than conventional vitamin D3 in protecting against inflammation [79].

However, AL-Serwi et al. (2021) [14] and M. El-Sherbiny et al. (2018) [79] used oral nano emulsion capsules to deliver Vitamin D3 in rats; our study utilized lipid nano capsules (LNCs)s, which are more effective for oral administration. Although negatively charged particles may initially be repelled by the similarly charged mucus layer in the gut, this can help prevent early uptake by non-target cells. Once these particles pass through the mucus, they can interact more efficiently with epithelial cells, leading to better absorption.

One of the major strengths of this study is its novel comparison between conventional and nano-formulated vitamin D3 in a controlled experimental model, which provides valuable insights into their differential effects on salivary gland histology and function. The use of multiple assessment tools—including histological, ultrastructural, and digital morphometric analyses—adds to the robustness and reliability of the findings. Additionally, the inclusion of biochemical parameters like serum calcium, insulin, and glucose levels allows for a more comprehensive evaluation of systemic effects.

Despite these strengths, several limitations should be acknowledged. First, the study was conducted on an animal model. It can restrict how far the results can be applied to people. Second, the sample size, while adequate for initial exploration, may not fully capture individual variability. Third, the study focused primarily on short-term outcomes; long-term effects of nano vitamin D3 supplementation on oral and systemic health remain unclear. Moreover, the potential toxicity or accumulation of nanoparticles over time was not addressed.

Future studies should aim to replicate these findings in larger cohorts and explore long-term safety and efficacy, particularly in clinical or human settings. Investigating the molecular mechanisms underlying the enhanced protective effects of nano vitamin D3 could provide further insight into its therapeutic potential. Additionally, exploring the effects of different nano-formulations, dosages, and delivery methods may help optimize treatment strategies. Finally, expanding the focus to include other salivary glands and tissues may help assess broader systemic implications of nano vitamin D3 supplementation.

## Conclusion

This study demonstrates the superior efficacy of vitamin D3-loaded lipid nano capsules over its conventional counterparts in protecting against calcium-deficiency-induced damage in parotid salivary glands. Rats treated with vitamin D3-loaded LNCs exhibited preserved glandular architecture, maintained secretory granule integrity, and improved biochemical profiles, indicating a more robust protective effect at both cellular and systemic levels. These findings suggest that vitamin D3-loaded lipid nano capsules offer a promising therapeutic strategy for mitigating oral and systemic complications associated with hypocalcemia.

## Data Availability

Data is provided within the manuscript or supplementary information files.
